# Cancer cell membrane-camouflaged biomimetic nanoparticles for enhancing chemo-radiation therapy efficacy in glioma

**DOI:** 10.7555/JBR.38.20240100

**Published:** 2024-05-30

**Authors:** Chunming Tang, Yanling Wang, Min Wu, Zhiji Wang, Yupeng Zhou, Ya Lin, Yijun Wang, Huae Xu

**Affiliations:** 1 Department of Pharmaceutics, School of Pharmacy, Nanjing Medical University, Nanjing, Jiangsu 211166, China; 2 Department of Pharmacy, the Second Affiliated Hospital of Nanjing Medical University, Nanjing, Jiangsu 210003, China

**Keywords:** glioblastoma multiforme, temozolomide, superparamagnetic iron oxide, chemo-radiation therapy, cancer cell membrane-coating

## Abstract

Glioblastoma multiforme (GBM) is a highly aggressive and lethal brain tumor with limited treatment options. To improve therapeutic efficacy, we developed a novel multifunctional nanoplatform, GM@P(T/S), comprised of polymeric nanoparticles coated with GBM cell membranes as well as co-loaded with temozolomide (TMZ) and superparamagnetic iron oxide (SPIO) nanoparticles. The successful preparation was confirmed in terms of particle size, morphology, stability, the *in vitro* drug release, and cellular uptake assays. We demonstrated that GM@P(T/S) exhibited the enhanced homotypic targeting, the prolonged blood circulation, and efficient blood-brain barrier penetration in both *in vitro* and *in vivo* studies. The combination of TMZ and SPIO nanoparticles within GM@P(T/S) synergistically improved chemo-radiation therapy, leading to a reduced tumor growth, an increased survival, and minimal systemic toxicity in the orthotopic GBM mouse models. Our findings suggest that GM@P(T/S) holds a great promise as a targeted and efficient therapeutic strategy for GBM.

## Introduction

Glioblastoma multiforme (GBM) is the most prevalent and malignant primary brain tumor in adults, with a median survival of only 12–15 months despite aggressive treatment strategies^[1–2]^. Standard care for GBM typically involves maximal surgical resection, followed by concurrent chemoradiotherapy with temozolomide (TMZ), an alkylating agent^[3]^. Accumulating studies indicate that combining radiation therapy and chemotherapy, known as chemoradiotherapy, holds significant synergistic effects in the treatment of GBM. Chemoradiotherapy capitalizes on the complementary action mechanisms of radiotherapy and chemotherapy. While radiation damages DNA and disrupts cell division, chemotherapy targets the rapidly dividing cancer cells, enhancing overall cytotoxic effects on tumor cells. The synergistic interaction between radiotherapy and chemotherapy leads to an increased tumor cell death and improved treatment outcomes, compared with either modality alone. Moreover, GBM is characterized by extensive intratumoral heterogeneity, with distinct subpopulations of cancer cells exhibiting varying levels of sensitivity to treatment. Combining radiotherapy and chemotherapy targets different tumor cell populations and overcomes resistance that may arise during treatment. This comprehensive approach increases the likelihood of eradicating both the bulk tumor and infiltrative cancer cells, thereby reducing the risk of disease recurrence^[4–7]^. However, the therapeutic efficacy of TMZ is often limited by its poor blood-brain barrier (BBB) penetration and the development of resistance mechanisms. Additionally, radiation therapy alone has shown limited success in achieving long-term survival because of the radioresistant nature of GBM cells and potential damage to surrounding healthy brain tissues^[8–9]^. Therefore, there is an urgent need for innovative therapeutic strategies that can improve drug delivery, enhance treatment efficacy, and minimize off-target toxicity.

Nanotechnology has revolutionized the field of cancer therapy by offering unique opportunities for the delivery, imaging, and theranostics of targeted drugs. Nanoparticles offer distinct advantages, such as a small size, a high surface area-to-volume ratio, and customizable physicochemical properties. These features enable efficient delivery of therapeutic agents to tumor sites while minimizing systemic toxicity^[10–12]^. Various nanocarriers, such as liposomes^[13]^, polymeric nanoparticles^[14]^, and inorganic nanoparticles^[15]^, have been explored for their potential to enhance the therapeutic outcomes of anticancer agents. The Food and Drug Administration (FDA)-approved poly(lactic-co-glycolic acid) (PLGA) stands out as an excellent drug carrier because of its biodegradability, biocompatibility, controlled drug release, and targeted delivery to tumor cells. Moreover, PLGA-based drug delivery systems offer the flexibility to incorporate multiple therapeutic agents, enabling combination therapy approaches to combat cancer through synergistic or complementary mechanisms. By co-encapsulating chemotherapeutic drugs with other agents, such as imaging contrast agents, photothermal agents, immunomodulators, or radiosensitizers, PLGA nanoparticles achieved multifunctionality and enhanced therapeutic outcomes through multimodal treatment strategies^[16–18]^. However, PLGA nanoparticles are prone to being intercepted by macrophages, leading to a diminished immunocompatibility. This interception hampers their capability to reach the target tissues, thereby compromising their therapeutic efficacy. Additionally, the recognition of PLGA nanoparticles by macrophages may trigger immune responses, leading to inflammation and other potential adverse reactions^[19–20]^.

As another type of nanoparticle, superparamagnetic iron oxide (SPIO) has garnered significant interest in the field of cancer therapy as a potential sensitizer for enhancing the efficacy of radiotherapy^[21]^. The main mechanism underlying the radiosensitizing effect of SPIO nanoparticles involves the generation of reactive oxygen species (ROS) upon exposure to ionizing radiation. When irradiated, SPIO nanoparticles induce the production of ROS through a process known as the Fenton reaction, wherein the catalytic properties of iron ions (Fe^2+^ or Fe^3+^) present in the nanoparticles facilitate the conversion of hydrogen peroxide (H_2_O_2_) into highly reactive hydroxyl radicals (•OH). These ROS induce DNA damage, protein oxidation, lipid peroxidation, and other oxidative stress-mediated cellular responses, ultimately leading to the enhanced cytotoxicity and apoptosis in cancer cells^[22–25]^. Continued research efforts to optimize SPIO-based radiosensitization strategies and translate them into clinical practice are warranted to realize their full potential in cancer therapy.

In recent years, cell membrane biomimetic drug delivery systems have emerged as a promising strategy in cancer therapy, leveraging the unique properties of natural cell membranes to enhance the targeted drug delivery and therapeutic efficacy. These biomimetic systems, particularly those using cancer cell membranes, have emerged as a promising strategy to enhance biocompatibility, stealth properties, and targeting capabilities^[26–28]^. Tumor cell membrane-coated nanoparticles may effectively evade immune recognition and clearance by the reticuloendothelial system, prolonging circulation time and improving tumor accumulation *via* homotypic targeting. Moreover, the inherent bioactivity of cancer cell membranes enables specific interactions with receptors overexpressed on cancer cells, facilitating efficient cellular internalization and payload delivery^[29–30]^.

Building upon these foundational concepts, the current study introduced a novel multifunctional platform using polymeric nanoparticles coated with cancer cell membrane [*i.e.*, GM@P(T/S)] to achieve precise therapeutic delivery for GBM treatment. By encapsulating both TMZ, a chemotherapy agent, and SPIO nanoparticles within the GM@P(T/S), we sought to capitalize on the synergistic effects of chemotherapy and radiotherapy. This approach aimed to thereby enhance therapeutic efficacy against GBM while addressing the inherent limitations of conventional treatment modalities.

## Materials and methods

### Materials

Temozolomide, coumarin 6 (C6), 2ʹ,7ʹ-dichlorodihydrofluorescein diacetate (DCFH-DA), and polyvinyl alcohol (PVA) were purchased from Aladdin (Shanghai, China). Tris(acetylacetonato)iron (Ⅲ), oleylamine, and benzyl ether were purchased from Energy Chemical (Shanghai, China). Poly(lactic-co-glycolic acid) (PLGA, 50/50-COOH, 15 K) was purchased from Shandong Medical Device Research Institute (Jinan, Shandong, China). Protease Inhibitor Cocktail (EDTA-Free, 100× in DMSO) was purchased from Selleck Chem (Houston, TX, USA). 4ʹ,6-diamidino-2-phenylindole (DAPI), cell counting kit-8 (CCK-8), and BCA protein assay kit were obtained from Beyotime (Shanghai, China). All other chemical reagents were of analytical grade and used without further purification.

### Cell lines and cell culture

The GBM U87MG, GL261, and RAW 264.7 cell lines were purchased from the American Type Culture Collection (ATCC, Manassas, VA, USA). For bioluminescent *in vivo* tumor imaging, we transduced the GL261 cells with a firefly luciferase-expressing lentivirus (GeneChem, Shanghai, China) according to the manufacturer's instructions. Briefly, the day before transduction, GL261 cells were seeded in a 96-well plate with 15000 cells per well. On the day of transduction, the medium was replaced by DMEM-containing lentivirus and incubated for 5 h. After transduction, the medium was replaced, and 48 h later, the cells were assayed for luciferase activity. Puromycin (Sigma-Aldrich, Bornem, Belgium) was used at 1 μg/mL once per week to select stably transduced cells. The above cell lines were cultured in DMEM medium supplemented with 10% (v/v) fetal bovine serum as well as 1% (v/v) penicillin and streptomycin at 37 ℃ with 5% CO_2_.

### Preparation of GM@P(T/S)

PLGA nanoparticles containing SPIO nanocrystals and TMZ were crafted using a refined version of the conventional oil-in-water single emulsion evaporation technique^[31]^. Specifically, 10 mg of PLGA, 3 mg of SPIO nanocrystals, and 1 mg of TMZ were dissolved in 1 mL of dimethylformamide, constituting the oil phase. The aqueous phase, comprising a 1% PVA solution presaturated with TMZ, was prepared to ensure saturation. This blend was then emulsified with 10 mL of the saturated aqueous phase by a probe-type sonicator operating at 150 W for 5 min, all the while maintained in an ice bath to preserve stability. Subsequently, the resulting suspension was gently stirred for 12 h at room temperature, allowing the organic solvent to evaporate completely. This was followed by centrifugation at 12000 *g* for 30 min to isolate the nanoparticles. The harvested P(T/S) nanoparticles were then washed three times with distilled water and resuspended in water. Additionally, C6 and Cy5 labeled PLGA nanoparticles were prepared by a similar process, substituting 0.1 wt% C6 or Cy5 for SPIO nanocrystals and TMZ.

The GBM U87MG or GL261 cell membrane was extracted through a hypotonic swelling procedure. Initially, the harvested cells were suspended in a hypotonic lysing buffer composed of 20 mmol/L Tris-HCl (pH 7.5), 10 mmol/L KCl, 2 mmol/L MgCl_2_, and a proteinase inhibitor cocktail. The GBM cells were disrupted using a Dounce homogenizer equipped with a snug-fitting pestle, ensuring 30 thorough passes. The homogenized mixture was then centrifuged at20000 *g* for 20 min at 4 ℃. The resulting pellet was discarded, and the supernatant was further centrifuged at 100000 *g* for 30 min at 4 ℃. Following centrifugation, the GBM membranes were isolated as the pellet and gently washed once with 1× phosphate-buffered saline (PBS) containing the proteinase inhibitor cocktail. The final pellet, now purified GBM membrane, was collected for subsequent experimental procedures.

To synthesize GM@P(T/S), a previously reported sonication technique was used^[32]^. Initially, the harvested GBM membranes were suspended in water and sonicated in a capped glass vial for 3 min, using a bath sonicator. This process yielded GBM membrane vesicles (GMVs). Subsequently, a mixture of P(T/S) and GMVs was prepared, maintaining a membrane protein to PLGA weight ratio of 1∶2. This mixture was sonicated using the bath sonicator for 5 min, resulting in the preparation of GM@P(T/S).

### Characterization of the GM@P(T/S)

The particle size and zeta potential of GMVs, P(T/S), and GM@P(T/S) were measured using dynamic light scattering (DLS; Nano ZS, Malvern, UK). The morphologies of P(T/S) and GM@P(T/S) were visualized by a transmission electron microscope (TEM, JEM-200CX, JEOL, Japan). For TEM analysis, the samples were prepared by depositing the nanoparticle droplets, at a concentration of 200 μg/mL, onto copper grids for 3 min. The excess droplets were then removed, and the grids were stained with 1% uranyl acetate for 30 s prior to imaging.

To quantify the drug-loading capacities of TMZ and SPIO nanocrystals, high-performance liquid chromatography (HPLC, LC-2010AHT, SHIMADZU, Japan) and inductively coupled plasma atomic emission spectrometry (ICP-AES, Optima 5300DV, PE, USA) were used, respectively. Briefly, 1 mg of the pre-prepared GM@P(T/S) was freeze-dried. The dried material was split into two equal parts: one for SPIO content analysis and the other for TMZ content determination. One portion, denoted as W_0_, was weighed and combined with 1 mL of acetone while being vortexed at 2000 rpm for 5 min. Subsequently, 1 mL of ultrapure water was added, mixed thoroughly, and centrifuged at 12000 *g* for 10 min. A total of 20 µL of the supernatant was extracted and injected into an HPLC system to ascertain the TMZ concentration (W). The following formula was used to compute the drug-loading efficiency (DL) of TMZ: DL_TMZ_ (%) = W/W_0_ × 100%. Next, the second portion, labeled as W_1_, was weighed and dissolved in a 3∶1 (v∶v) mixture of concentrated hydrochloric acid and concentrated nitric acid. Once fully dissolved, the solution was diluted with ultrapure water. The iron ion concentration was measured *via* ICP-AES to determine the SPIO content (W_2_). Finally, the drug-loading efficiency of SPIO was calculated using the formula: DL_SPIO_ (%) = W_2_/W_1_ × 100%.

The average particle sizes of P(T/S) and GM@P(T/S) were evaluated in both 1× PBS and 100% fetal bovine serum (FBS) at various time points, ranging from 24 h to 14 days at 37 ℃, to assess their stability over time.

For the *in vitro* release study of TMZ, 0.5 mL of either P(T/S) or GM@P(T/S) was mixed with 0.5 mL of FBS and placed in a dialysis tube (3.5K MWCO) submerged in 50 mL of PBS buffer solution (pH 7.4) containing 0.2% Tween-80. At specific time intervals, a 500 µL aliquot of the solution outside the dialysis bag was withdrawn for HPLC analysis to monitor the release profile of TMZ.

### Homologous targeting ability of GM@P(T/S)

To assess the *in vitro* targeting effects of GM@P(T/S) on U87MG or GL261 cells, these cells were initially seeded in confocal dishes, 24-well plates, or 6-well plates and cultured for 24 h. Subsequently, the cells were incubated with 100 μg/mL of C6-loaded P(T/S) and GM@P(T/S) for 1 h. After incubation, the cells in the confocal dishes were washed with PBS twice, stained with DAPI, fixed with 4% paraformaldehyde (PFA), and then imaged using a confocal laser scanning microscope (CLSM; LSM 880, Zeiss, Jena, Thuringia, Germany) to visualize any targeting effects. Flow cytometry was performed for further quantitative analysis. Briefly, cells in the 24-well plates were detached using a trypsin-EDTA solution, washed twice with media, and then analyzed using a flow cytometer (AccuriC6, BD, Franklin Lakes, NJ, USA) to measure the fluorescence intensity associated with the targeted nanoparticles. To determine the iron (Fe) uptake by U87 MG or GL261 cells, the cells in the 6-well plates were lysed by adding 0.5 mL of 1% Tween-80 to each well. The resulting cell lysate from each well was mixed with 1 mL of nitric acid. The mixtures were allowed to stand at room temperature for 24 h, followed by annealing at 80 ℃ for 8 h to remove the acid. Finally, the samples were resuspended in distilled water and analyzed using ICP-AES to quantify the Fe content.

### Evaluation of the *in vitro* immune escape

RAW 264.7 cells (1 × 10^5^ cells/dish) were seeded in confocal dishes, and cultured for 24 h. Subsequently, the cells were incubated with C6-loaded P(T/S) and GM@P(T/S) for 4 h. After the incubation, the cells in the confocal dishes were washed three times with PBS, stained with DAPI, fixed with 4% PFA, and imaged using a CLSM.

### *In vitro* combined antitumor effect

To evaluate the *in vitro* therapeutic effect of GM@P(T/S) on U87MG or GL261 cells, we performed CCK-8 assays. The cells were plated in 96-well plates and treated with P(T/S), GM@P(T/S), as well as a combination of GM@P(T/S) and X-ray irradiation (3 Gy) for 24 h or 48 h. Additionally, different concentrations of TMZ (ranging from 10 to 400 μg/mL) were tested. Subsequently, 10 μL of CCK-8 solution (5 mg/mL) was added to each well. After a 4-h incubation, the absorbance was measured at a wavelength of 450 nm using a microplate reader. Cells grown without any treatment (0 concentration) served as the control group.

### *In vitro* ROS generation ability

U87MG cells were treated with GM@P(T/S) for 2 h in a confocal culture dish. After removing the free GM@P(T/S), DCFH-DA (25 mmol/L) was added. Following a 30-minute incubation, the cells were washed three times with PBS before undergoing X-ray irradiation at a dose of 3 Gy. For laser confocal imaging, an excitation wavelength of 488 nm was used, and emissions were captured in the 500–600 nm wavelength range.

### Penetration in U87MG multicellular spheroids

To investigate the penetration capabilities of GM@P(T/S) within multicellular spheroids, U87MG cells were seeded into a PrimeSurface^TM^ 96-well plate, with 2 × 10^3^ cells per well. After allowing the cells to proliferate for 72 h, the multicellular spheroids reached a diameter of approximately 500 μm. Subsequently, these spheroids were incubated with C6-loaded P(T/S) and GM@P(T/S) for an additional 4 h. Following incubation, the spheroids were washed and fixed using 4% paraformaldehyde to preserve their structure. To visualize the distribution of C6 within the spheroids, Z-stack imaging was performed using a CLSM. This technique captured images at 10 μm intervals, spanning from the bottom to the middle of the spheroids, enabling a detailed analysis of the fluorescence emitted by C6. This approach allowed us to observe the penetration abilities of GM@P(T/S) within the complex multicellular environment of the spheroids.

### Evaluation of the *in vitro* BBB permeability

An *in vitro* BBB model was established using a transwell cell culture system, including both endothelial cells (hCMEC/D3) and astrocytes (HA1800). Initially, HA1800 cells were seeded at a density of 2 × 10^5^ cells per well on the underside of the transwell chamber and allowed to adhere for 24 h. Subsequently, with utmost precision, the chamber was inverted, and hCMEC/D3 cells were seeded at a density of 1 × 10^5^ cells per well, topping the chamber. The integrity of the cellular monolayer was evaluated by measuring transepithelial electrical resistance (TEER) values using a MillicellERS voltmeter (Millipore). Only monolayers with TEER values exceeding 300 Ω cm^2^ were selected as the BBB model for transmigration studies. Next, C6-labeled P(T/S) and GM@P(T/S) nanoparticles were introduced into the upper chamber at a concentration of 10 μg/mL, while the lower chamber was filled with FBS-free medium. Following incubation periods of 2 h, 12 h, and 24 h, the C6 fluorescence of the supernatant in the upper chamber and the medium in the lower chamber were analyzed using a microplate reader. The transport ratio in each compartment was calculated based on the initial amount of Cy5-loaded nanoparticles introduced, ensuring accurate quantification of nanoparticle translocation across the BBB model.

### *In vivo* pharmacokinetics

Healthy Sprague Dawley (SD) rats, weighing between 180 and 220 grams, were used in a pharmacokinetic study. Following the intravenous injection of P(T/S) and GM@P(T/S) (2.5 mg TMZ equivalent per kilogram and 20 mg SPIO equivalent per kilogram), blood samples of 500 μL were collected from the retro-orbital sinus of the rats at various time points (0.083, 0.167, 0.25, 0.5, 1, 2, 4, 8, 12, and 24 h). Immediately after collection, the blood samples were measured and dissolved in a lysis buffer (1 mL, containing 1% Triton X-100) with brief sonication. To determine the TMZ concentration in blood, methanol (1 mL) was added to each blood sample, and the mixture was incubated overnight at −20 ℃. The samples were then vortexed and centrifuged at 3500 *g* for 20 min. The TMZ concentration in the supernatant was quantified using HPLC. To quantify the Fe concentration, 1 mL of nitric acid was added to each blood sample. The samples were left at room temperature for 24 h, followed by annealing at 80 ℃ for 8 h to remove the acid. Finally, the samples were resuspended in distilled water for the determination of Fe content using ICP-AES.

### Orthotopic glioma model for brain tumors

Female C57BL/6 mice, aged between six and eight weeks, were obtained from the Comparative Medicine Center of Yangzhou University. All animal experiments adhered strictly to the Guide for the Care and Use of Laboratory Animals, endorsed by China Pharmaceutical University. An orthotopic glioma model was established following the protocol outlined in our previous report^[33]^. Briefly, the mice were anesthetized and injected with 2.0 × 10^5^ GL261-Luc cells into the right striatum of the brain. These cell lines were genetically modified to emit a luciferase signal. The injection of GL261-Luc cells was administered using a total volume of 5 μL of physiological saline, delivered at a controlled rate of 0.2 μL per minute. After the injection, the scalp was sutured, and the mice were closely monitored postoperatively to ensure their return to normal activity. Tumor growth was tracked using an *in vivo* image system (IVIS, PerkinElmer, Waltham, MA, USA).

### *In vivo* imaging

GL261-Luc tumor-bearing mice were randomly assigned to three groups and intravenously administered with either saline, Cy5-labeled P(T/S), or Cy5-labeled GM@P(T/S) at a Cy5 dose of 30 nmol/kg. Sequential images were captured using the IVIS system at designated time intervals (0, 2, 4, 8, 12, and 24 h) after injection. Furthermore, the brains of the mice were harvested at each time point for *in vivo* imaging. The fluorescence intensities of the regions of interest were quantitatively analyzed using Living Image Software.

### Biodistribution of GM@P(T/S)

A single intravenous dose of GL261 cell membrane-coated GM@P(T/S) and naked P(T/S) nanoparticles, suspended in 200 μL of saline (2.5 mg TMZ/kg, 20 mg SPIO/kg), was administered *via* the tail vein to mice with orthotopic GL261-luc glioblastoma tumors. Four hours after injection, the tumor-bearing mice were humanely euthanized, and their vital organs, including the heart, spleen, lung, liver, kidney, and brain, were meticulously excised, thoroughly washed, dried, and accurately weighed. To quantify the delivery of TMZ and SPIO to the tumor, the GBM tumor was delicately dissected from the brain. Both the tumor tissues and major organs were homogenized in 1 mL of 1% Triton X-100 using a sonic homogenizer operating at 100 kHz for three minutes. Subsequently, the concentrations of TMZ and SPIO were determined using the previously validated methods.

### *In vivo* anti-tumor efficacy

On the 10th day following implantation, GL261 orthotopic GBM-bearing mice were randomly divided into seven groups, each containing six mice. The mice were intravenously administered with GM@P(T/S) + R, GM@P (S) + R, GM@P (T), GM@P(T/S), saline + R, free TMZ, or saline, every two days for a total of five doses. Immediately after each injection, the mice were anesthetized, and their tumor luminescence intensity was meticulously tracked using the Lumina IVIS Ⅲ system. The relative photon flux was standardized to the baseline intensity, represented as F/F0, where F0 indicated the bioluminescence intensity on day 10. Additionally, the body weight of each mouse was measured every two days, and a Kaplan-Meier survival curve was constructed to evaluate survival rates over time.

### Statistical analysis

Data were presented as the mean ± standard deviation. To assess statistically significant differences, we performed either a two-tailed unpaired Student's *t*-test or a one-way/two-way analysis of variance (ANOVA), followed by Tukey's multiple comparisons test. All statistical analyses were conducted using GraphPad Prism software, with significance set at *P* < 0.05.

## Results

### Preparation and characterization of GM@P(T/S)

**Figure 1:**
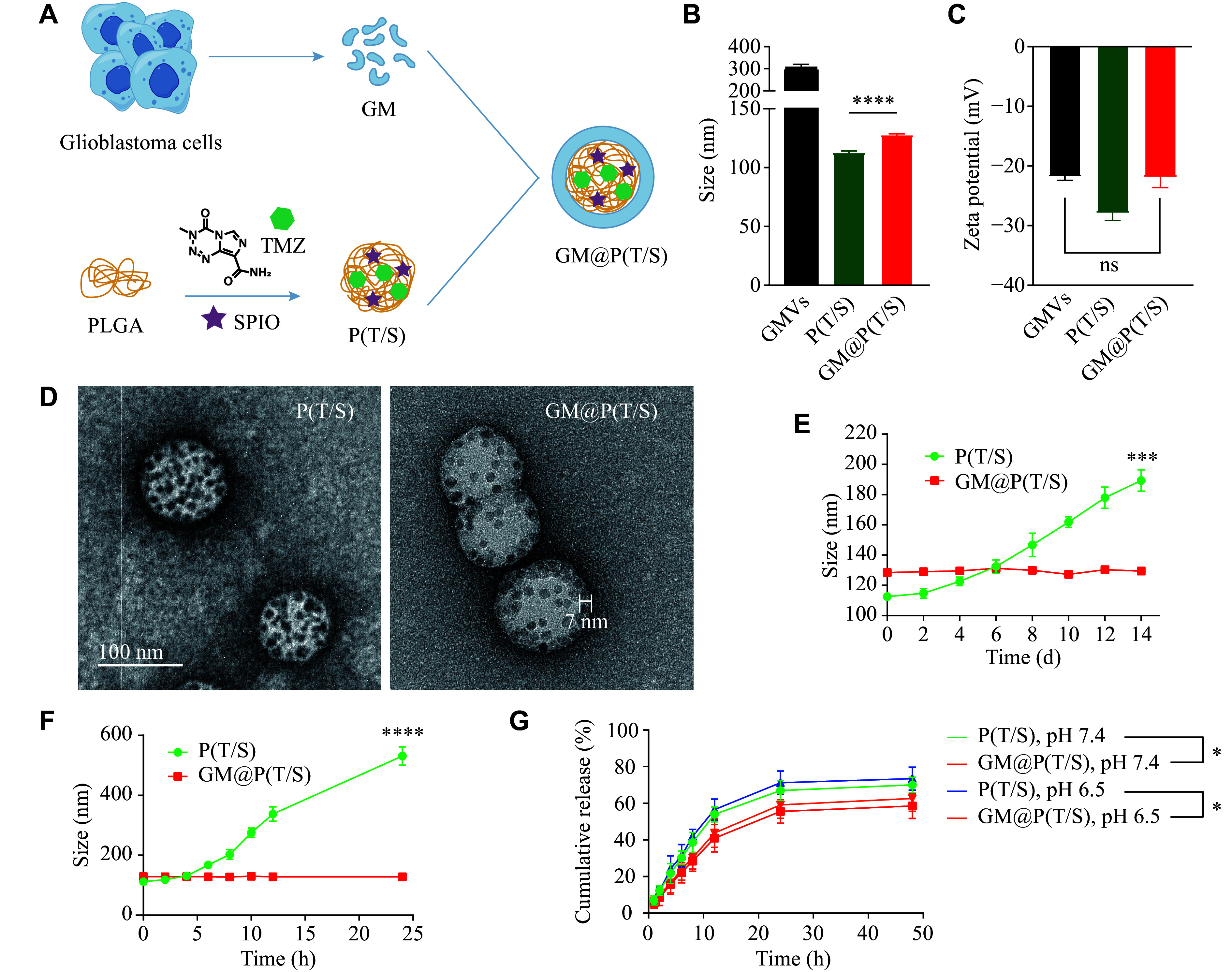
Fabrication and characterization of GM@P(T/S). A: Schematic illustration of cancer cell membrane-camouflaged SPIO and TMZ-encapsulated polymeric nanoparticles [GM@P(T/S)]. B and C: Particle sizes and zeta potentials of GMVs, P(T/S) and GM@P(T/S), respectively. D: TEM images of P(T/S) and GM@P(T/S). Scale bar, 100 nm. E and F: Long-term stability of P(T/S) and GM@P(T/S). Size changes of these nanoparticles in PBS (E) and 100% fetal bovine serum (F) over time were measured. G: *In vitro* release of TMZ from P(T/S) and GM@P(T/S) in 50% fetal bovine serum. Error bars indicate standard deviation (*n* = 3). The significance was determined by the Student's *t*-test. ^*^P < 0.05, ^***^P < 0.001, and ^****^P < 0.0001. Abbreviations: TMZ, temozolomide; PLGA, poly(lactic-co-glycolic acid); SPIO, superparamagnetic iron oxide; GM, glioma cell membrane; P(T/S), PLGA nanoparticles co-loaded with TMZ and SPIO; GM@P(T/S), glioma cell membrane-coated PLGA nanoparticles co-loaded with TMZ and SPIO; GMVs, glioma membrane vesicles; ns, not significant.

As illustrated in ***Fig. 1A***, the GM@P(T/S) comprised PLGA nanoparticles co-loaded with TMZ and SPIO as the inner core [P(T/S)], with a coating shell consisting of the glioma cell membrane (GM). To fabricate the P(T/S), we initially synthesized oleylamine-modified SPIO *via* a high-temperature reductive decomposition method, as described in our previous studies, resulting in particles with a narrow size distribution and excellent superparamagnetism^[34]^.

The mean hydrodynamic diameter and Zeta potential of P(T/S) and GM@P(T/S) were determined using DLS. P(T/S) exhibited a mean hydrodynamic diameter of 113.1 nm and a mean Zeta potential of −28.0 mV (***Fig. 1B*** and ***1C***). Following encapsulation with the GM, GM@P(T/S) showed an average particle size of 128.2 nm and a surface potential of −22.0 mV (***Fig. 1B*** and ***1C***). The increased size compared with P(T/S) and the similar surface charge compared with GMVs indicated the successful coating of the GM onto P(T/S). Furthermore, TEM images showed a core-shell structure of GM@P(T/S), displaying a unilamellar membrane over their polymeric cores (***Fig. 1D***). The drug-loading capacities of SPIO and TMZ in GM@P(T/S) were 22.6% (± 0.45%) and 2.85% (± 0.27%), respectively, as measured using ICP-AES and HPLC (***Supplementary Table 1***, available online). The reduction in drug loading, compared with P(T/S), similarly demonstrates the successful coating of GM in GM@P(T/S).

To assess the colloidal stability of GM@P(T/S), we monitored the size changes of these nanoparticles in both PBS and 100% FBS over time. GM@P(T/S) exhibited superior colloidal stability, compared with P(T/S) (***Fig. 1E*** and ***1F***), indicating a protective effect conferred by the GM coating. This enhanced stability suggests that GM@P(T/S) may possess improved biocompatibility and prolonged circulation times *in vivo*, potentially leading to more effective therapeutic outcomes. Moreover, the delayed release of TMZ from GM@P(T/S) in both pH 7.4 and pH 6.5 buffer solutions suggested that the GM might serve as a diffusion barrier, slowing down the drug diffusion rate (***Fig. 1G***).

### Homotypic targeting effect of GM@P(T/S)

**Figure 2:**
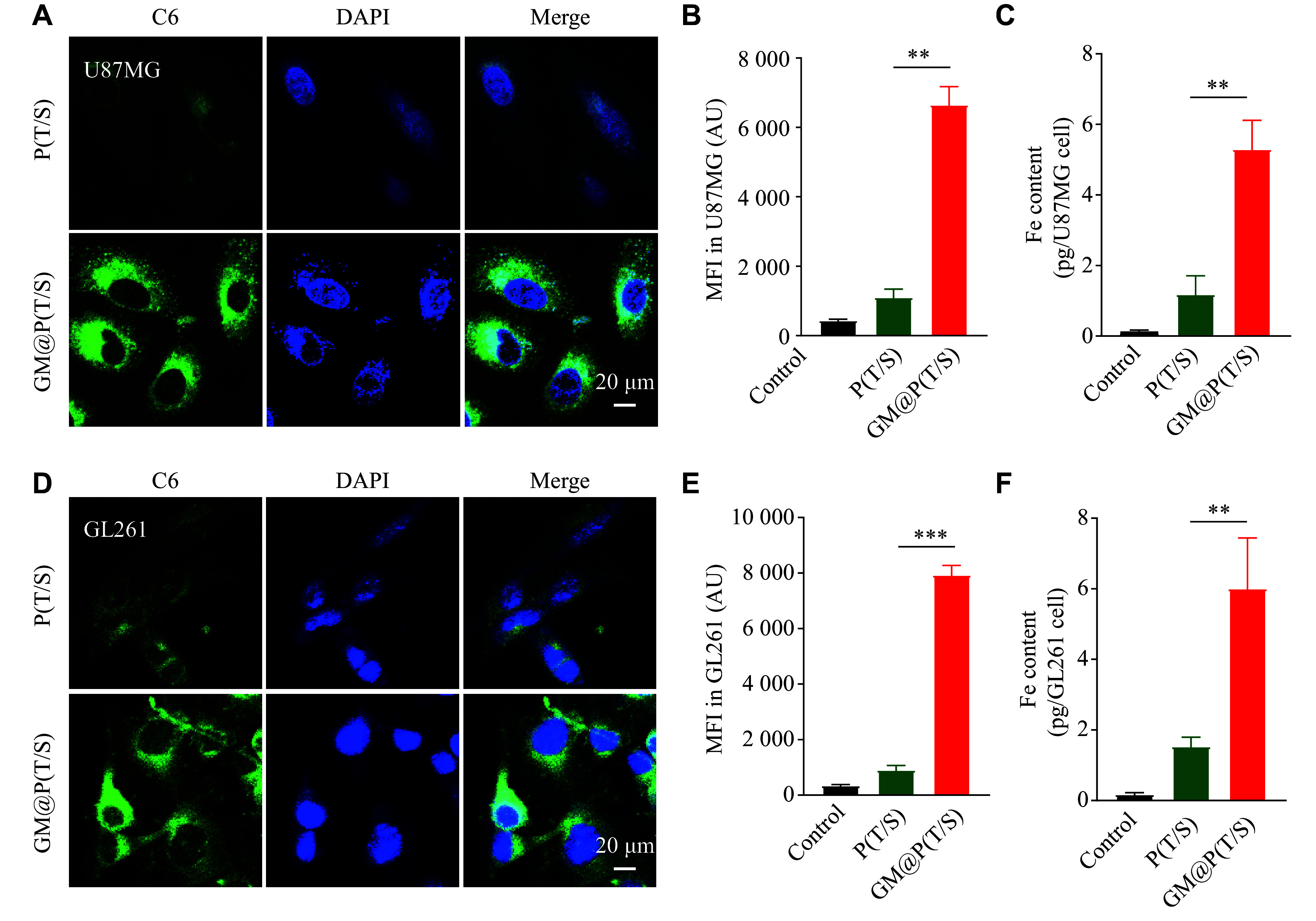
GM@P(T/S) as a homotypically targeted delivery vehicle. A: Fluorescent imaging of U87MG cells incubated with bare P(T/S), and GM@P(T/S) coated with membrane derived from U87MG cells. All nanoparticles were loaded with C6 (green). The nucleus was stained with DAPI (blue). B: Binding of fluorescently labeled bare P(T/S) and GM@P(T/S) to U87MG glioma cells, as analyzed by flow cytometry. C: Quantitative analysis of Fe uptake of various nanoparticles by U87MG cells. D: Fluorescent imaging of GL261 cells incubated with bare P(T/S) and GM@P(T/S) coated with membrane derived from GL261 cells. All nanoparticles were loaded with C6 (green). The nucleus was stained with DAPI (blue). E: Binding of fluorescently labeled bare P(T/S) and GM@P(T/S) to GL261 glioma cells, as analyzed by flow cytometry. F: Quantitative analysis of Fe uptake of various nanoparticles by U87MG cells. Error bars indicate standard deviation (*n* = 3). The significance was determined by one-way ANOVA with Tukey's correction. ^**^P < 0.01 and ^***^P < 0.001. Abbreviations: AU, arbitrary unit; C6, coumarin 6; MFI, mean fluorescence intensity; P(T/S), PLGA nanoparticles co-loaded with TMZ and SPIO; GM@P(T/S), glioma cell membrane coated PLGA nanoparticles co-loaded with TMZ and SPIO.

To demonstrate the capability of GM@P(T/S) in homotypically targeting cancer cells for drug delivery purposes, we extracted membranes from U87MG or GL261 cells and coated them onto PLGA cores loaded with C6. Fluorescence microscopy revealed that incubating GM@P(T/S) with cultured U87MG cells *in vitro* led to a significantly enhanced uptake, compared with uncoated P(T/S) (***Fig. 2A***). Then we performed flow cytometric analysis to quantify this discrepancy in the uptake, and found that the U87MG membrane coating facilitated approximately a 6.0-fold increase in uptake relative to uncoated P(T/S) (***Fig. 2B***; ***Supplementary Fig. 1A***, available online). Furthermore, quantitative analysis using ICP-AES also showed that the GM@P(T/S) group exhibited the highest uptake, aligning with the fluorescence images and flow cytometric analysis (***Fig. 2C***). The intracellular uptake of GM@P(T/S) was further assessed in GL261 glioma cells, and the results similar to that in U87 MG cells were observed (***Fig. 2D***–***2F***; ***Supplementary Fig. 1B***, available online). These findings demonstrated that the GM comprising the outer shell of GM@P(T/S) substantially enhanced the cellular uptake of P(T/S).

To assess the antiphagocytic properties of GM@P(T/S), we used CLSM to visualize its intracellular uptake by mouse macrophage RAW264.7 cells, and found that the GM@P(T/S) group showed a lower level of macrophage uptake, compared with the P(T/S) group (***Supplementary Fig. 2***, available online). This finding may be primarily attributed to the presence of CD47 on the membranes of GBM, which suppressed the uptake of macrophage cells within the reticular endothelial system.

### *In vitro* anticancer activity

**Figure 3:**
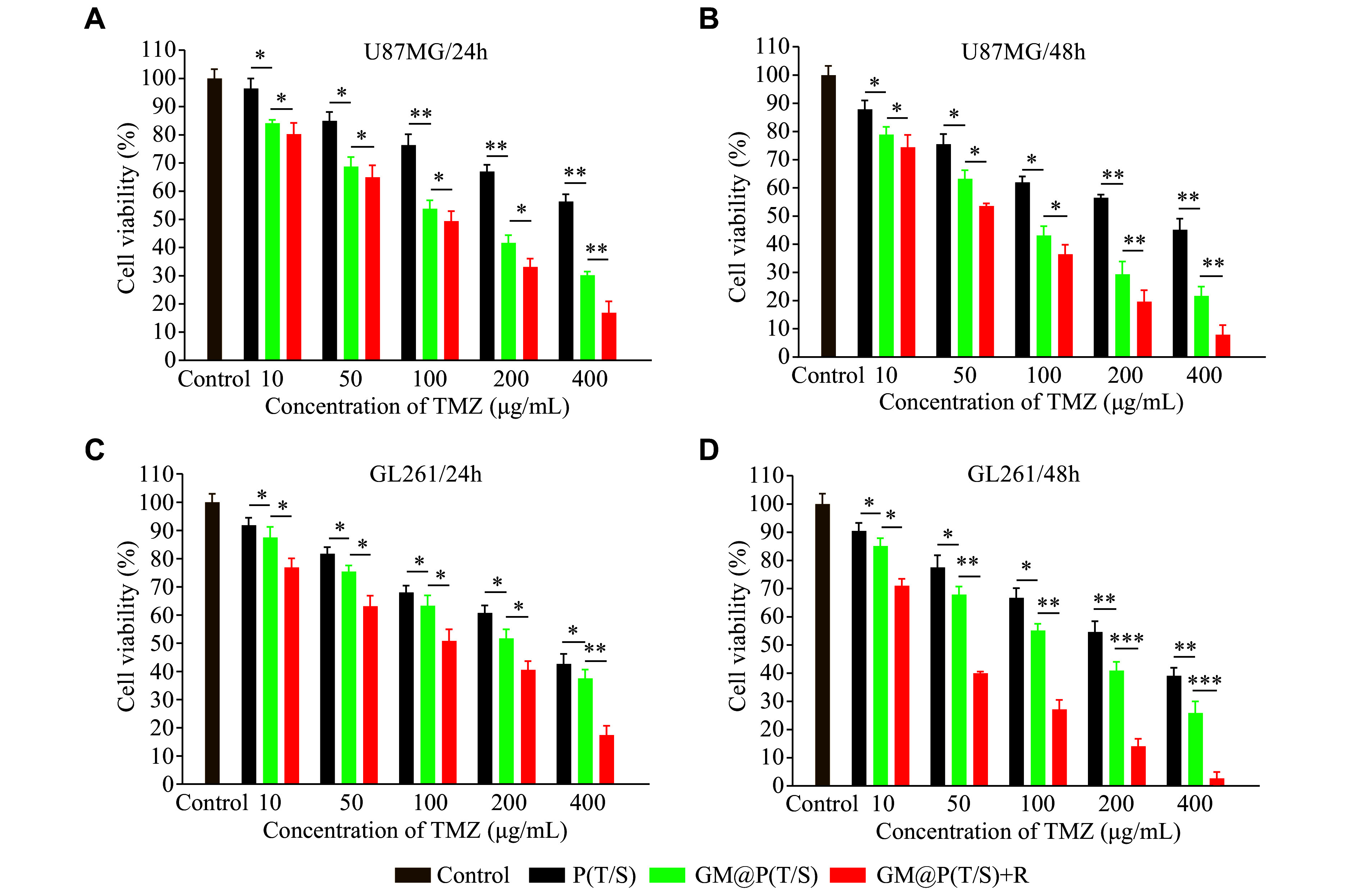
*In vitro* antitumor efficacy of GM@P(T/S). A and B: *In vitro* cytotoxicity of P(T/S), GM@P(T/S), and GM@P(T/S) coupled with X-ray at various concentrations of TMZ on U87MG cells after 24 h (A) or 48 h (B) incubation. C and D: *In vitro* cytotoxicity of P(T/S), GM@P(T/S), and GM@P(T/S) coupled with X-ray at various concentrations of TMZ on GL261 cells after 24 h (C) or 48 h (D) incubation. Cells grown without any treatment served as the control group. Error bars indicate standard deviation (*n* = 6). The significance was determined by one-way ANOVA with Tukey's correction. ^*^P < 0.05, ^**^P < 0.01, and ^***^P < 0.001. Abbreviations: TMZ, temozolomide; P(T/S), PLGA nanoparticles co-loaded with TMZ and SPIO; GM@P(T/S), glioma cell membrane-coated PLGA nanoparticles co-loaded with TMZ and SPIO; R, radiotherapy.

SPIO has been extensively used as a radiosensitizer to enhance radiotherapy^[35]^. Consequently, we delved into the potential of combining chemotherapy with radiotherapy using GM@P(T/S) against U87MG and GL261 cells. ***Fig. 3A*** and ***3B*** depict the viability of U87MG cells after incubation with varying concentrations of TMZ in the P(T/S), GM@P(T/S), and GM@P(T/S) + R [*i.e.*, GM@P(T/S) coupled with an X-ray exposure of 3 Gy] groups for 24 and 48 h. Similarly, ***Fig. 3C*** and ***3D*** present the viability of GL261 cells under identical conditions. Notably, GM@P(T/S) showed a pronounced cytotoxic effect on cancer cells in a dose- and time-dependent pattern. Even at low concentrations, the GM@P(T/S) group demonstrated comparable or superior cytotoxicity, compared with the uncoated P(T/S) group. As the incubation time progressed, the cytotoxic differences between the P(T/S) and GM@P(T/S) groups became increasingly evident. Moreover, the survival rate of cells was significantly lower in the GM@P(T/S) + R group than in the GM@P(T/S) group. Furthermore, the estimated half maximal inhibitory concentration (IC_50_) value was significantly lower in the GM@P(T/S) + R group than in the GM@P(T/S) group (***Supplementary Table 2***, available online).

The generation of ROS by GM@P(T/S) under X-ray irradiation may be the key factor to enhance therapeutic effects. Consequently, we examined the intracellular ROS generation ability in U87 MG cells using DCFH-DA as a ROS probe. As shown in ***Supplementary Fig. 3*** (available online), when U87MG cells were treated with both GM@P(T/S) and X-ray irradiation, strong green fluorescence of DCF was observed, which was approximately 7.0 and 4.1 times higher than that of the groups treated with GM@P(T/S) or X-ray irradiation alone, respectively. This indicated a high intracellular ROS production from GM@P(T/S) coupled with X-ray exposure. Notably, the X-ray irradiation alone also increased green fluorescence to some extent (***Supplementary Fig. 3***).

These findings suggest that the combined approach of GM@P(T/S) and irradiation may suppress the proliferative capacity of both cell types, thereby enhancing the potential anticancer activity for GBM therapy *via* ROS generation.

### *In vitro* BBB traversal and tumor penetration by GM@P(T/S)

**Figure 4:**
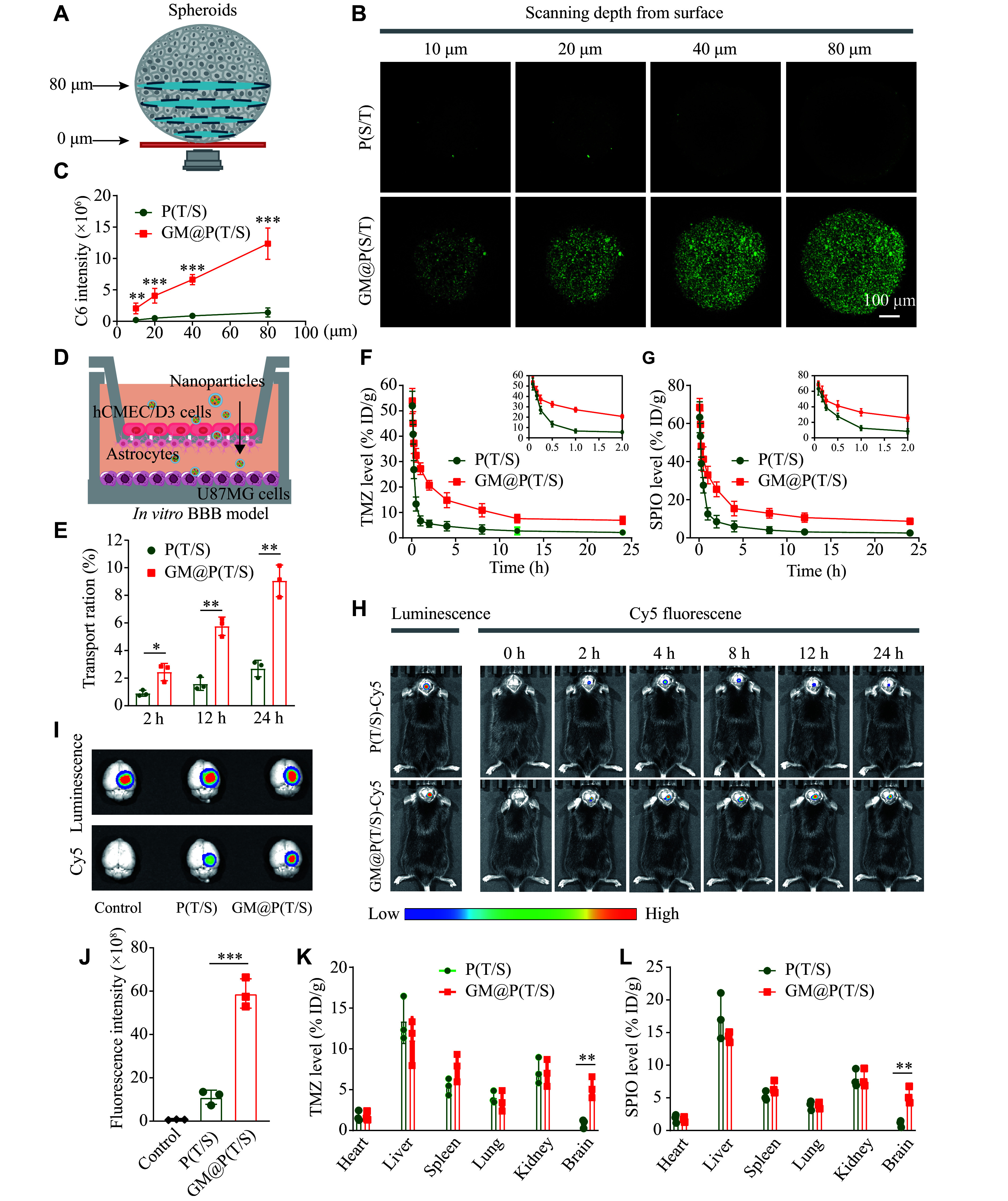
Prolonged blood circulation, enhanced BBB penetration, and brain tumor accumulation mediated by biomimetic GM@P(T/S). A: Schematic illustration of the *in vitro* 3D spherical tumor model. B and C: Penetration and quantification of C6 loaded GM@P(T/S) distribution in U87MG multicellular spheroids after 4 h of incubation. D: Illustration of the *in vitro* BBB model. E: Transport ratios of bare P(T/S) and GM@P(T/S) in the hCMEC/D3 monolayer of the *in vitro* BBB model. F and G: Pharmacokinetic behavior of bare P(T/S) and GM@P(T/S) in rats after intravenous administration of different formulations at the TMZ (F) dose of 2.5 mg/kg or SPIO (G) dose of 20 mg/kg. The inset shows the enlarged drug-time curve of the first six time points. H: *In vivo* imaging in mice bearing orthotopic GL261-Luc glioblastoma tumor following injection of Cy5-labeled P(T/S) and GM@P(T/S) at a Cy5 dose of 20 nmol/kg (*n* = 3). I: Bioluminescence and Cy5 fluorescence images of excised GBM-bearing brains taken from GL261-Luc bearing mice 24 h after receiving the treatments (*n* = 3). J: Quantitative analysis of Cy5 fluorescence intensity in excised mouse brains. K and L: Quantification of TMZ (K) and SPIO (L) accumulation in different organs and tumors excised from GL261-Luc bearing mice at 4 h post-injection of P(T/S) and GM@P(T/S) (2.5 mg TMZ equivalent per kilogram, 20 mg SPIO equivalent per kilogram). Error bars indicate standard deviation (*n* = 3). The significance was determined by Student's *t*-test (C, E, K, and L) or one-way ANOVA with Tukey's correction (J). ^*^P < 0.05, ^**^P < 0.01, and ^***^P < 0.001. Abbreviations: BBB, blood-brain barrier; ID/g, injected dose per gram; Cy5, cyanine 5; P(T/S), PLGA nanoparticles co-loaded with TMZ and SPIO; GM@P(T/S), glioma cell membrane coated PLGA nanoparticles co-loaded with TMZ and SPIO.

Despite some evidence indicating a high homotypic targeting capacity of GM@P(T/S), its ability to penetrate the BBB and reach deep cerebral GBM cells remains inconclusive. In the current study, we assessed the tumor penetration efficacy of GM@P(T/S) in U87MG multicellular spheroids (***Fig. 4A***). Notably, even at a scanning depth of 10 μm, the uncoated P(T/S) failed to produce a notable fluorescence, which was primarily confined to the periphery of the multicellular spheroids (***Fig. 4B***). Conversely, fluorescence generated by GM@P(T/S) treatment was distinctly visible within the multicellular spheroids, even at a scanning depth of 80 μm, and this was quantitatively verified (***Fig. 4C***). These findings further underscore the remarkable tumor penetration capability of GM@P(T/S).

To assess the transendothelial potential of GM@P(T/S), we subsequently seeded U87MG cells onto the bottom layer of the transwell setup (***Fig. 4D***), and observed a significant enhancement in the cellular uptake of GM@P(T/S) by U87MG cells, compared with P(T/S) (***Fig. 4E***). Unsurprisingly, P(T/S) lacking the cancer cell membrane coating exhibited a significantly low permeability through the endothelial monolayer, because of the tight junctions between adjacent cells.

Taken together, these findings indicate that GM@P(T/S) may be capable of traversing BBB and reaching deep-seated cerebral GBM cells.

### Pharmacokinetics and biodistribution of GM@P(T/S)

Before evaluating the therapeutic potential of GM@P(T/S) i*n vivo*, we initially investigated its pharmacokinetics in healthy mice. Specifically, we monitored the blood levels of TMZ and SPIO to estimate the plasma clearance kinetics following a single intravenous injection through the tail vein. As shown in ***Fig. 4F***, ***4G*** and ***Supplementary Table 3*** (available online), GM@P(T/S) demonstrated an extended blood circulation time, compared with the unmodified P(T/S). This enhanced circulation duration suggests that the membrane camouflaging technique may help nanoparticles evade immune recognition and subsequent elimination from the bloodstream. This effect may be attributed to the presence of 'self' recognizable proteins on the membrane, as previously reported^[36]^.

Given the enhanced pharmacokinetics of GM@P(T/S) achieved through homologous camouflaging, we anticipated that this would facilitate its accumulation within the tumor. Thus, we investigated the brain-targeting ability of GM@P(T/S) *in vivo*. We treated C57/BL6 mice bearing orthotopically implanted luciferase expressing GL261 (GL261-Luc) cells with Cy5-labeled GM@P(T/S), and observed a significant accumulation of the nanoparticles in the brain, as evidenced by the intense Cy5 fluorescence detected at 2 h post-injection, which persisted up to 24 h (***Fig. 4H***; ***Supplementary Fig. 4***, available online). In contrast, Cy5-labeled P(T/S) exhibited significantly less accumulation in the brain. *Ex vivo* imaging further demonstrated the brain targeting capacity of GM@P(T/S) (***Fig. 4I*** and ***4J***).

Subsequently, we evaluated the biodistribution of GM@P(T/S) camouflaged with the GL261 cell membrane in mice with orthotopic GL261 tumors. The concentration of delivered TMZ in the tumor (approximately 5.22% ID/g) was slightly lower than that in the liver and comparable to that in the spleen and kidney. This concentration was significantly higher than in other organs, including the heart and lung (***Fig. 4K***). Conversely, a much lower accumulation of TMZ in the tumor (approximately 0.86% ID/g) was observed for the uncoated P(T/S). Similarly, SPIO showed a comparable accumulation profile in the tumor and major tissues to that of TMZ, with a tumor uptake of 5.35% ID/g in mice treated with GM@P(T/S) (***Fig. 4L***).

These findings indicate that GM@P(T/S) may not only possess an extended blood circulation time but also exhibit a strong homotypic tumor-targeting capability.

### Combinational *in vivo* anti-tumor activity of GM@P(T/S)

**Figure 5:**
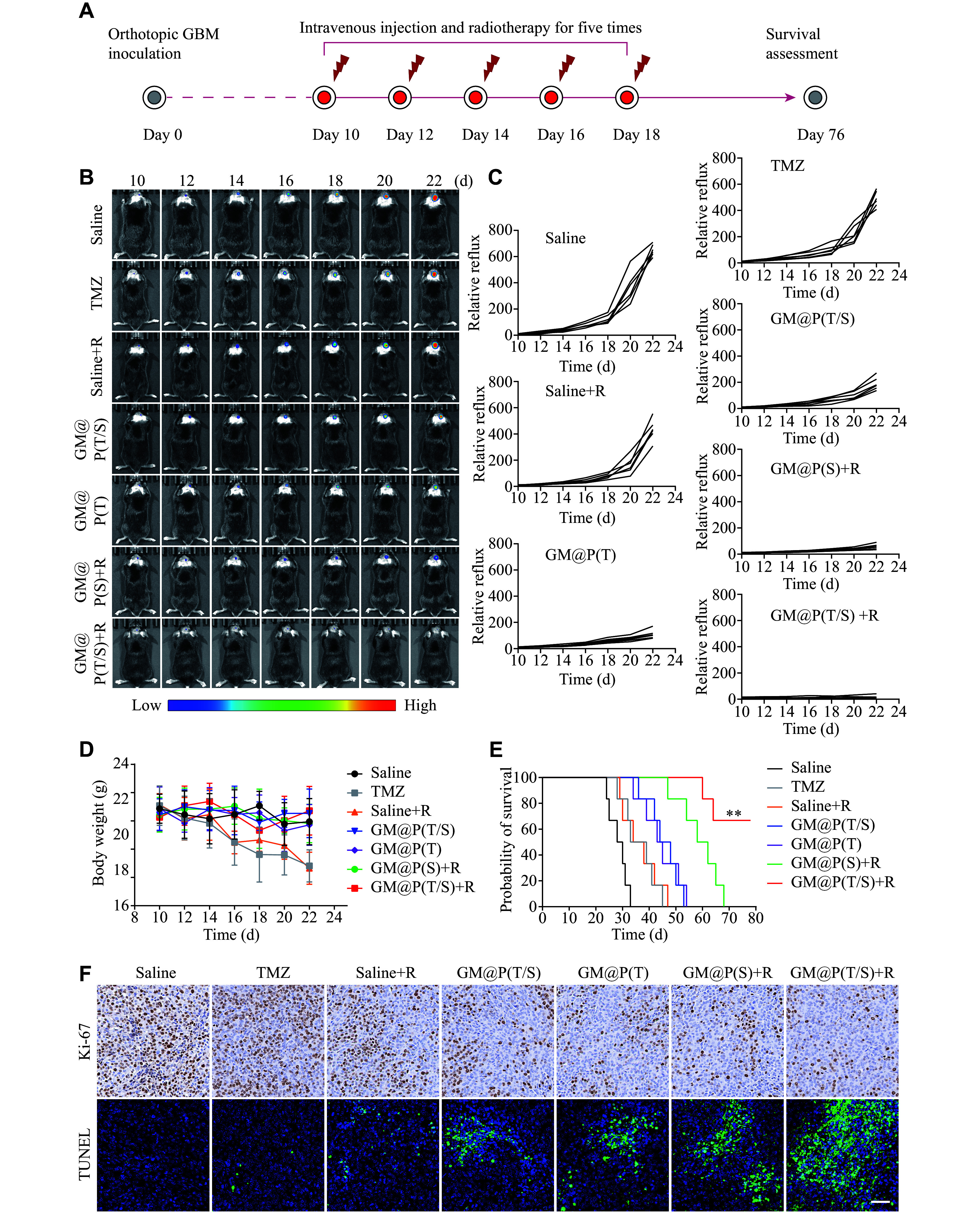
Combinational *in vivo* antitumor activity of GL261 cell membrane-camouflaged GM@P(T/S) in the orthotopic GL261-Luc glioma mouse model. A: A schematic diagram depicts the timeline of the efficacy study. B: The growth of GBM tumors in mice, monitored through bioluminescence imaging, following treatments with GM@P(T/S) + R (2.5 mg/kg TMZ + 20 mg/kg SPIO), GM@P(S) + R (20 mg/kg SPIO), GM@P(T) (2.5 mg/kg TMZ), GM@P(T/S) (2.5 mg/kg TMZ + 20 mg/kg SPIO), saline + R, free TMZ (2.5 mg/kg TMZ), or saline alone. The mice were administered the treatments on days 10, 12, 14, 16, and 18. C: Mean GL261-Luc tumor luminescence levels of mice following different treatments. D: Body weight changes of mice resulting from the different treatments up to day 22. Error bars indicate standard deviation (*n* = 6). E: Kaplan–Meier survival of mice receiving different treatments (*n* = 6). F: Immunohistochemistry in tumor tissues from mice following different treatments. Scale bar, 50 μm. Error bars indicate standard deviation (*n* = 6). The significance was determined using the log-rank (Mantel-Cox) test (E). ^**^P < 0.01. Abbreviations: GBM, glioblastoma multiforme; TMZ, temozolomide; R, radiotherapy; GM@P(T), glioma cell membrane coated PLGA nanoparticles loaded with TMZ; GM@P(S), glioma cell membrane coated PLGA nanoparticles loaded with SPIO; GM@P(T/S), glioma cell membrane coated PLGA nanoparticles co-loaded with TMZ and SPIO; TUNEL, terminal deoxynucleotidyl transferase-mediated dUTP nick end labeling.

The therapeutic potential of GL261 cell membrane-camouflaged GM@P(T/S) was investigated in mice with orthotopic GL261-Luc glioblastoma. The mice were treated with a cycle of five doses of different formulations: GM@P(T/S) + R (2.5 mg/kg TMZ + 20 mg/kg SPIO), GM@P(S) + R (20 mg/kg SPIO), GM@P(T) (2.5 mg/kg TMZ), GM@P(T/S) (2.5 mg/kg TMZ + 20 mg/kg SPIO), saline + R, free TMZ (2.5 mg/kg), or saline (***Fig. 5A***). To monitor tumor growth, D-luciferin was injected into the mice, and the *in vivo* bioluminescence imaging was observed. The results showed that GM@P(T/S) + R treatment was the most effective in suppressing GBM tumor growth, demonstrated by a significant reduction in luciferase bioluminescence, nearly eliminating tumor growth (***Fig. 5B***). Quantitative bioluminescence analysis corroborated these findings and further demonstrated the significant inhibition of GL261-Luc levels in the mouse brains achieved by GM@P(T/S) + R treatment (***Fig. 5C***). Notably, mice receiving GM@P(T/S) + R treatment exhibited minimal weight loss, indicating the relatively low toxicity of this therapeutic approach. In contrast, mice treated with free TMZ or saline + R exhibited significant weight loss, reflecting severe systemic side effects (***Fig. 5D***). Furthermore, survival curve analysis showed that GM@P(T/S) + R treatment significantly prolonged the lifespan of mice up to 76 days, a substantial increase compared with other control groups (***Fig. 5E***). We subsequently analyzed the histology of tumor tissues. Compared with the control groups, mice treated with GM@P(T/S) + R showed not only the lowest Ki-67-positive cell ratios in their tumor tissues but also exhibited the highest number of apoptotic tumor cells, indicated by TUNEL staining, upon immunohistochemical analysis (***Fig. 5F***; ***Supplementary Fig. 5***, available online). Taken together, these findings highlight that GM@P(T/S) may mediate safe and enhanced chemo-radiation therapy in mice bearing orthotopic brain tumors.

### Safety of GM@P(T/S)

**Figure 6:**
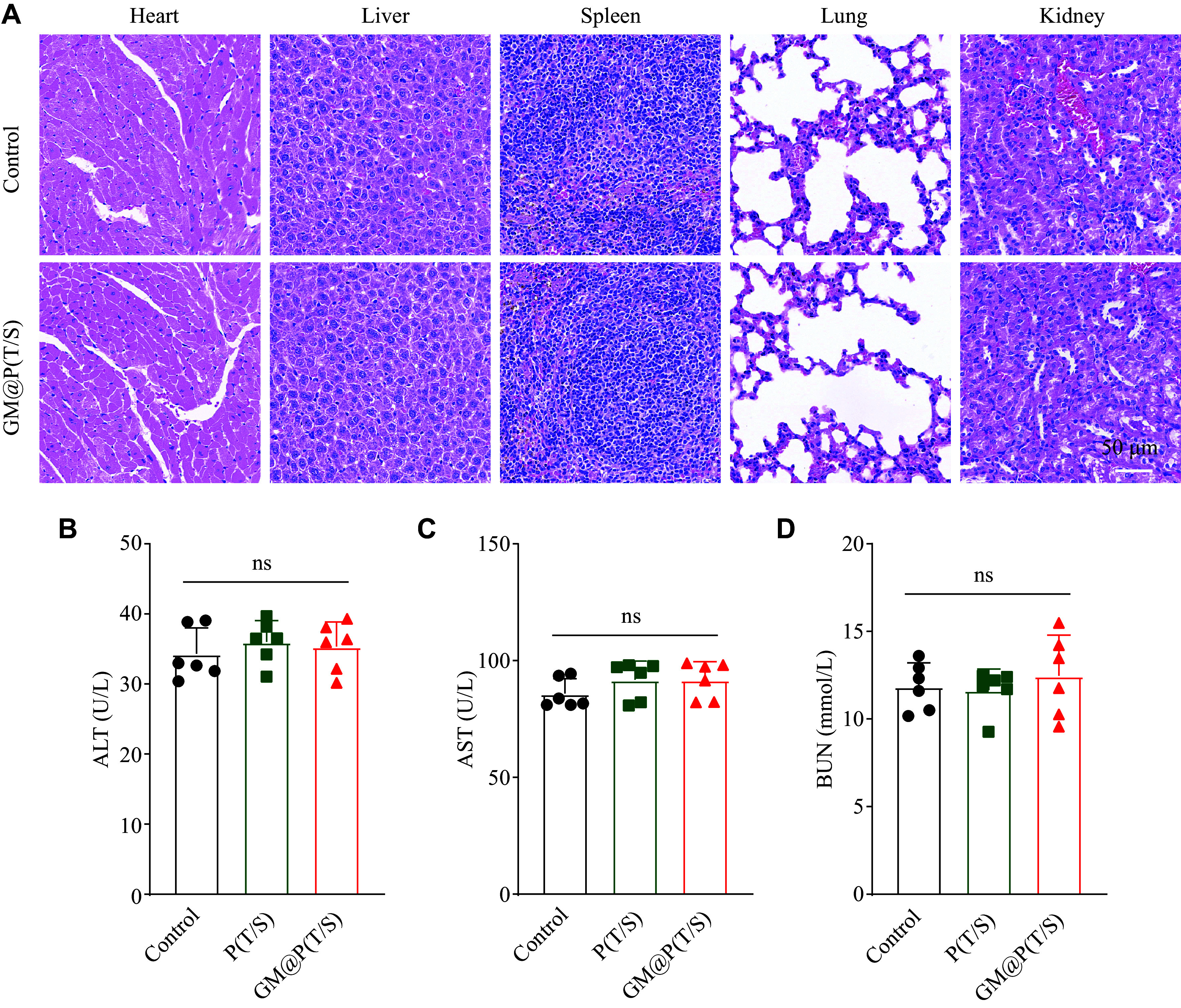
Safety evaluation of GM@P(T/S). A: Hematoxylin and eosin staining of main organs, including the heart, liver, spleen, lung, and kidney, from mice treated with GM@P(T/S) or controls. B–D: Hematological analysis of the mice in each group at 22 days post-transplantation. Error bars indicate standard deviation (*n* = 6). The significance was determined by one-way ANOVA with Tukey's correction. Abbreviations: ALT, alanine transaminase; AST, aspartate transaminase; BUN, blood urea nitrogen; ns, not significant; P(T/S), PLGA nanoparticles co-loaded with TMZ and SPIO; GM@P(T/S), glioma cell membrane coated PLGA nanoparticles co-loaded with TMZ and SPIO.

The *in vivo* biosafety remains a paramount concern in nanomedicine. To assess the biocompatibility of GM@P(T/S), mice that received either saline or GM@P(T/S) treatment were euthanized upon the completion of the treatment period, and major organs and blood samples were collected for hematoxylin and eosin histological and hematological analysis, respectively. Notably, no obvious pathological changes were found in the main organs between the control and GM@P(T/S) groups, indicating the good biocompatibility of the used nanomaterials (***Fig. 6A***). In addition, no significant differences were observed in the hematological parameters across different groups at 22 days post-transplantation (***Fig. 6B–6D***). These findings indicate that GM@P(T/S) exhibits satisfactory biocompatibility, paving the way for promising clinical applications.

## Discussion

**Figure 7:**
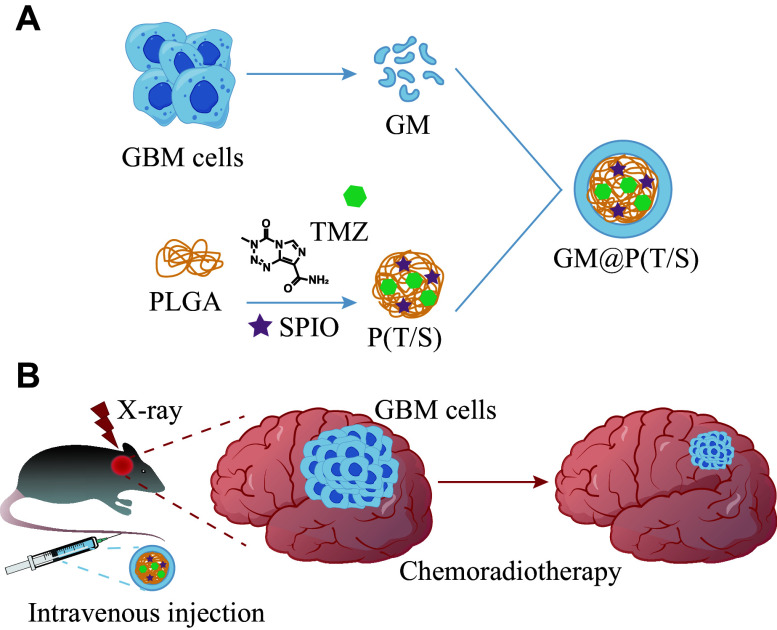
Preparation of a biomimetic PLGA-based drug co-delivery system GM@P(T/S) for the enhanced chemo-radiation therapy of glioma. The inner core of GM@P(T/S) is composed of PLGA nanoparticles co-loaded with temozolomide (TMZ), a chemotherapy agent, and superparamagnetic iron oxide (SPIO), a radiosensitizer. The biomimetic outer shell of GM@P(T/S) is derived from the membranes of GBM cells and uses the native biocompatibility and homotypic target ability. When coupled with X-ray exposure, GM@P(T/S) inhibits the growth of cancer cells by a synergistic effect. Abbreviations: GBM, glioblastoma multiforme; TMZ, temozolomide; PLGA, poly(lactic-co-glycolic acid); SPIO, superparamagnetic iron oxide; GM, glioma cell membrane; P(T/S), PLGA nanoparticles co-loaded with TMZ and SPIO; GM@P(T/S), glioma cell membrane coated PLGA nanoparticles co-loaded with TMZ and SPIO.

GBM remains a formidable malignancy, presenting significant treatment challenges because of its infiltrative nature, remarkable heterogeneity, and resistance to conventional therapeutic approaches. Despite aggressive interventions, including maximal surgical resection combined with chemoradiotherapy using TMZ, the prognosis for GBM patients remains dismal, with a median survival of only 12 to 15 months^[37–38]^. Given the intricate etiopathogenesis of GBM, there is an urgent need for innovative therapeutic approaches that may enhance drug delivery, augment treatment efficacy, and minimize collateral toxicity. Nanotechnology has emerged as a game-changer in cancer therapy, offering the targeted delivery and reduced systemic toxicity^[39]^. Nanoparticles, such as PLGA and SPIO, possess unique properties that make them ideal candidates for drug delivery systems. PLGA-based nanoparticles offer controlled drug release and precise targeting capabilities, while SPIO nanoparticles enhance radiotherapy efficiency through radiosensitization^[19]^. Drawing inspiration from the inherent ability of cancer cells to traverse the BBB and localize with homologous cells, known as homotypic targeting, we have designed and developed a novel GBM cancer cell membrane-cloaked polymeric nanoparticle system, designated as GM@P(T/S) (***Fig. 7***). This innovative nanoparticle system uses PLGA nanoparticles co-loaded with TMZ and SPIO as its core, aiming to achieve effective chemo-radiation therapy in glioma. By exploiting the natural tropism of cancer cells, GM@P(T/S) offers a targeted and effective therapeutic approach with minimal off-target toxicity that potentially facilitates the GBM treatment.

The GM@P(T/S) platform we developed exhibits promise in addressing the challenges of GBM treatment. Firstly, a key advantage of the GM@P(T/S) is its ability to evade immune recognition and clearance by the reticuloendothelial system, significantly prolonging circulation time and enhancing tumor accumulation. By incorporating cancer cell membranes, the nanoparticles may engage in specific interactions with overexpressed receptors on cancer cells, thereby facilitating efficient cellular internalization *via* receptor-mediated endocytosis and targeted payload delivery. This targeted approach not only enhances therapeutic agent accumulation at tumor sites but also significantly reduces off-target effects on healthy tissues, ultimately mitigating systemic toxicity and improving overall treatment outcomes. Secondly, the synergistic combination of TMZ and SPIO nanoparticles encapsulated within the GM@P(T/S) platform holds great promise for enhancing cytotoxicity against GBM cells. TMZ specifically targets rapidly dividing cancer cells, while SPIO nanoparticles sensitize the tumors to radiotherapy by inducing the generation of ROS. This combined therapeutic strategy may effectively overcome limitations correlated with traditional treatment modalities, offering a personalized approach tailored to the unique needs of GBM patients.

However, despite the promising benefits of the GM@P(T/S) platform, several challenges remain to be addressed. Scalability and reproducibility of the manufacturing process for cancer cell membrane-coated nanoparticles are crucial considerations. Standardized protocols are essential to ensure consistent particle size, membrane integrity, and drug loading efficiency, which are vital for effective and safe therapeutic outcomes. Furthermore, rigorous preclinical evaluations are imperative to assess the long-term stability and safety of these nanoparticles *in vivo*. Although this refined approach to GBM therapy has the potential to address the unmet clinical needs of patients by combining the targeted delivery and synergistic therapy to improve treatment outcomes but minimize systemic toxicity, future studies are needed to optimize this strategy and facilitate its translation into clinical practice. It is also crucial to address the manufacturing challenges and conduct comprehensive preclinical studies to ensure the safety and efficacy of the GM@P(T/S) platform in GBM treatment.

In summary, the current study provides valuable insights into GBM therapy by presenting an innovative and comprehensive strategy to address the limitations of the current regimens of GBM treatment. Through integrating polymeric nanoparticles coated with cancer cell membranes and loaded with both TMZ and SPIO nanoparticles, our approach has the promise of enhancing the effectiveness of chemoradiotherapy. This strategy could pave the way for personalized and precise treatment approaches in the management of GBM.

## SUPPLEMENTARY DATA

Supplementary data to this article can be found online.
