# Supplementary material for: Cancer cell membrane-camouflaged biomimetic nanoparticles for enhancing chemo-radiation therapy efficacy in glioma
Source: J Biomed Res. 2024 May 30;39(1):87–102. doi: 10.7555/JBR.38.20240100 (PMC11873587; doi:10.7555/JBR.38.20240100)
Supplement: Supplementary file 1 — Supplementary data to this article can be found online. [file jbr-39-1-87-S1.pdf]

Available online at www.jbr-pub.org.cn

Open Access at PubMed Central

Journal of Biomedical Research, 2025 39(1): 1–3

Supplementary Data

JBR

Cancer cell membrane-camouflaged biomimetic nanoparticles for enhancing chemo-radiation therapy efficacy in glioma

Chunming Tang[1,△], Yanling Wang[1,△], Min Wu[1], Zhiji Wang[1], Yupeng Zhou[1], Ya Lin[1], Yijun Wang[2,✉], Huae Xu[1,✉]

[1]*Department of Pharmaceutics, School of Pharmacy, Nanjing Medical University, Nanjing, Jiangsu 211166, China;* [2]*Department of Pharmacy, the Second Affiliated Hospital of Nanjing Medical University, Nanjing, Jiangsu 210003, China.*

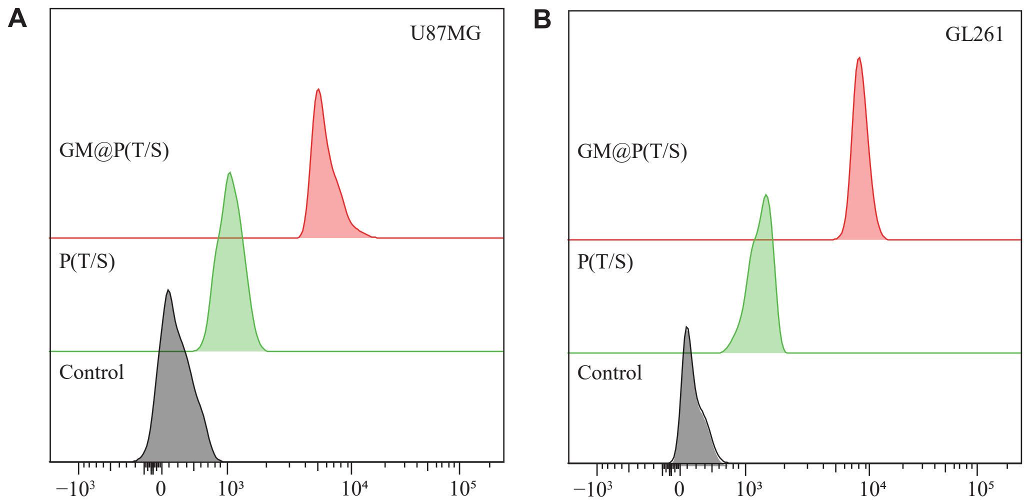

Supplementary Fig. 1 **Evaluation of homologous targeting ability.** Binding of fluorescently labeled bare P(T/S) and GM@P(T/S) to U87MG glioma cells (A) and GL261 glioma cells (B), as analyzed by flow cytometry.

△These authors contributed equally to this work.

✉Corresponding authors: Yijun Wang, Department of Pharmacy, the Second Affiliated Hospital of Nanjing Medical University, 121 Jiangjiayuan Road, Nanjing, Jiangsu 210003, China. E-mail: efywyj@163.com; Huae Xu, Department of Pharmaceutics, School of Pharmacy, Nanjing Medical University, 818 East Tianyuan Road, Nanjing, Jiangsu 211166, China. E-mail: xuhuae@njmu.edu.cn.

Received: 08 April 2024; Revised: 22 May 2024; Accepted: 24 May 2024; Published online: 30 May 2024

CLC number: R943, Document code: A

The authors reported no conflict of interests.

This is an open access article under the Creative Commons Attribution (CC BY 4.0) license, which permits others to distribute, remix, adapt and build upon this work, for commercial use, provided the original work is properly cited.

© 2025 by Journal of Biomedical Research.

https://doi.org/10.7555/JBR.38.20240100

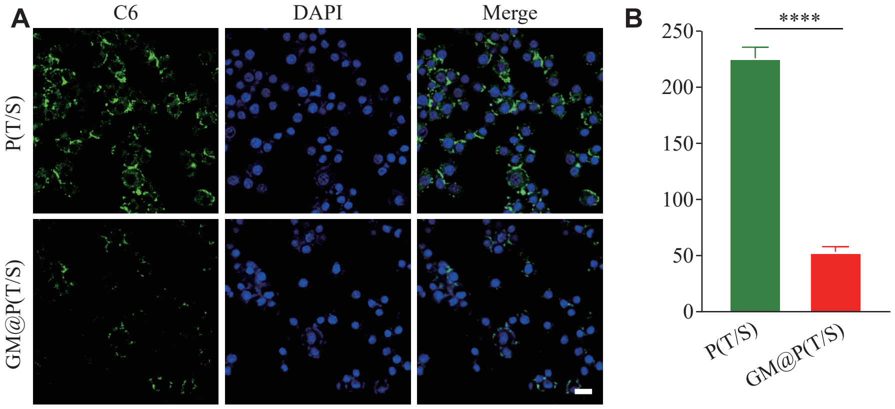

Supplementary Fig. 2 **Evaluation of immune escape capability.** Representative fluorescent images (A) taken by a fluorescence microscope and intensity quantification (B) for C6 uptake in RAW264.7 cells one hour post incubation with P(T/S) and GM@P(T/S). The nucleus was stained with DAPI (blue). The P(T/S) and GM@P(T/S) were labeled with C6 (green). Scale bars, 20 μm. Error bars indicate standard deviation (n = 3). The significance was determined by one-way ANOVA with Tukey's correction. $^{****}P < 0.000\,1$.

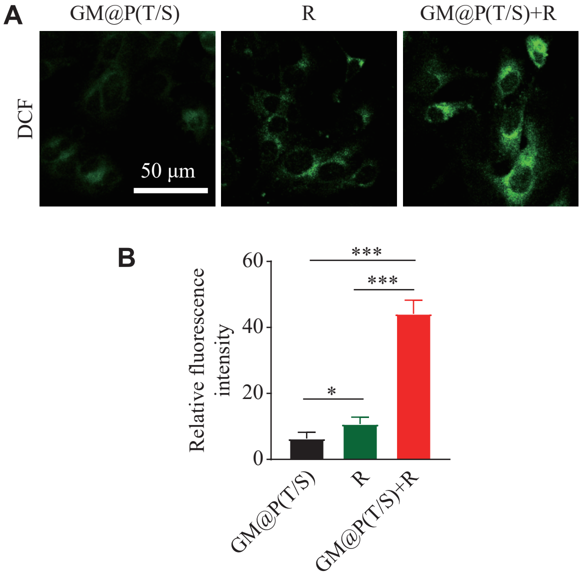

Supplementary Fig. 3 **Evaluation of ROS generation ability.** Representative fluorescent images (A) taken by a fluorescence microscope and intensity quantification (B) for 2′,7′-dichlorodihydrofluorescein diacetate (DCFH-DA) staining in U87MG cells following treatments with GM@P(T/S), R, and GM@P(T/S) + R. Scale bars, 50 μm. Error bars indicate standard deviation (n = 3). The significance was determined by one-way ANOVA with Tukey's correction. $^{*}P < 0.05$ and $^{***}P < 0.001$.

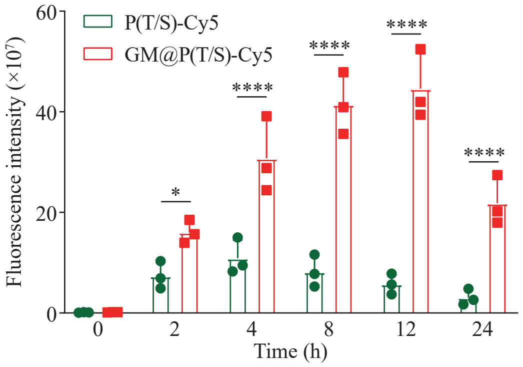

Supplementary Fig. 4 **ROI of fluorescent intensities from brain area at six time points.** Error bars indicate standard deviation (n = 3). The significance was determined by Student's t-test. $^{*}P < 0.05$ and $^{****}P < 0.000\,1$.

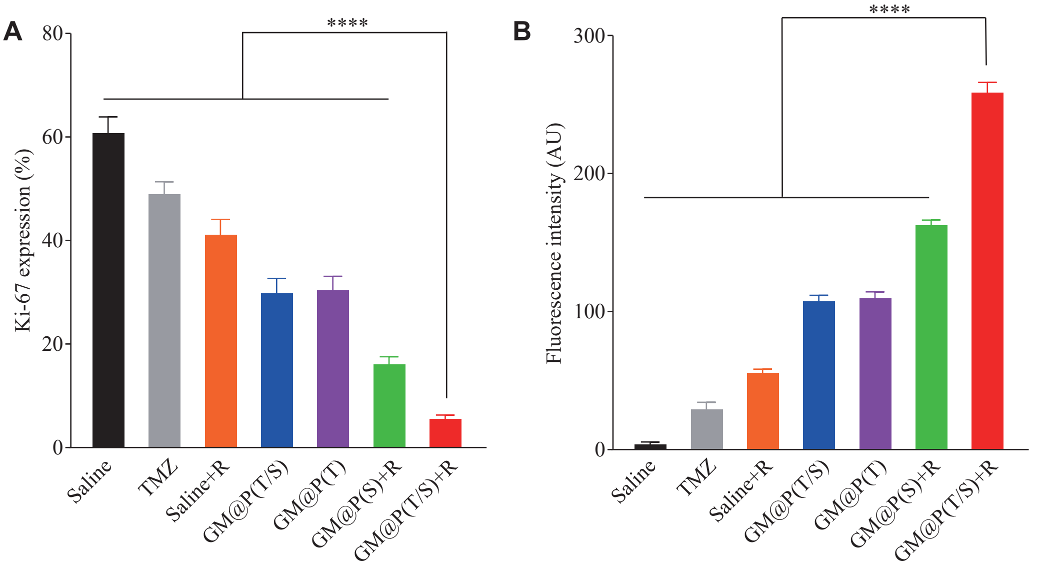

Supplementary Fig. 5 **Quantitative analysis of the Ki-67 levels (A) and TUNEL signal area (B).** Error bars indicate standard deviation (n = 6). The significance was determined by one-way ANOVA with Tukey's correction. $^{****}P < 0.0001$.

Supplementary Table 1 **Drug loading and entrapment efficiency capacity of P(T/S) and GM@P(T/S)**

	DL (TMZ, %)	EE (TMZ, %)	DL (SPIO, %)	EE (SPIO, %)
P(T/S)	3.74±0.32	83.2±2.15	30.2±0.68	94.3±1.24
GM@P(T/S)	2.85±0.27*	82.7±1.66	22.6±0.45****	94.0±1.05

Data are presented as mean ± standard deviation (n = 3). The significance was determined by Student's t-test, compared with the P(T/S) group. $^{*}P < 0.05$ and $^{****}P < 0.000\,1$. Abbreviations: DL, drug loading; EE, entrapment efficiency.

Supplementary Table 2 **The IC_{50} of P(T/S) and GM@P(T/S) against U87MG and GL261 cells**

	24 h		48 h	
	U87	GL261	U87	GL261
P(T/S)	409.3±30.2	287.8±24.0	246.5±21.0	216.7±28.7
GM@P(T/S)	134±6.3$^{####}$	168.8±14.7$^{####}$	86.2±4.0$^{####}$	114.7±7.3$^{####}$
GM@P(T/S)+R	75.5±10.1***	97.4±10.8****	54.0±3.5**	27.83±1.9****

Data are presented as mean ± standard deviation (n = 6). One-way ANOVA was used to identify significant differences between the treatment groups. Statistical significance between the groups GM@P(T/S) and GM@P(T/S)+R: $^{**}P < 0.01$, $^{***}P < 0.001$, $^{****}P < 0.0001$; Statistical significance between the groups P(T/S) and GM@P(T/S): $^{####}P < 0.0001$.

Supplementary Table 3 **Pharmacokinetic parameters of TMZ after intravenous administration of P(T/S) and GM@P(T/S) to rats at the TMZ dose of 2.5 mg/kg**

Parameters	P(T/S)	GM@P(T/S)
Cmax (mg/L)	7.8±1.1	8.2±1.3
$t_{1/2}$ (h)	3.5±0.7	9.7±0.8
$AUC_{(0-\infty)}$ (mg ·h/L)	18.1±3.8	56.2±5.3
$MRT_{(0-\infty)}$ (h)	5.2±0.5	20.1±1.4
CL (mL/h/kg)	258.5±51.2	66.8±8.5

Data are presented as mean ± standard deviation (n = 3). The significance was determined by Student's t-test. $^{**}P < 0.01$, $^{***}P < 0.001$, and $^{****}P < 0.000\,1$.
